# Paper-Based Detection Device for Alzheimer’s Disease—Detecting β-amyloid Peptides (1–42) in Human Plasma

**DOI:** 10.3390/diagnostics10050272

**Published:** 2020-04-30

**Authors:** Wei-Hsuan Sung, Jung-Tung Hung, Yu-Jen Lu, Chao-Min Cheng

**Affiliations:** 1 Chang Gung Memorial Hospital Linkou Medical Center, Taoyuan 33305, Taiwan; w.h.sung0109@gmail.com; 2 College of Medicine, Chang Gung University, Taoyuan 33302, Taiwan; 3 Institute of Stem Cell & Translational Cancer Research, Chang Gung Memorial Hospital Linkuo Medical Center, Taoyuan 33305, Taiwan; felixhjt@gmail.com; 4 Department of Neurosurgery, Chang Gung Memorial Hospital Linkou Medical Center, Taoyuan 33305, Taiwan; 5 Institute of Biomedical Engineering, National Tsing Hua University, Hsinchu 30013, Taiwan

**Keywords:** Alzheimer’s disease, β-amyloid peptide, paper-based ELISA, P-ELISA, point of care testing

## Abstract

The diagnosis of Alzheimer’s disease (AD) is frequently missed or delayed in clinical practice. To remedy this situation, we developed a screening, paper-based (P-ELISA) platform to detect β-amyloid peptide 1–42 (Aβ42) and provide rapid results using a small volume, easily accessible plasma sample instead of cerebrospinal fluid. The protocol outlined herein only requires 3 μL of sample per well and a short operating time (i.e., only 90 min). The detection limit of Aβ42 is 63.04 pg/mL in a buffer system. This P-ELISA-based approach can be used for early, preclinical stage AD screening, including screening for amnestic mild cognitive impairment (MCI) due to AD. It may also be used for treatment and stage monitoring purposes. The implementation of this approach may provide tremendous impact for an afflicted population and may well prompt additional and expanded efforts in both academic and commercial communities.

Alzheimer’s disease (AD) is one the most common irreversible neurodegenerative diseases across the globe. The massive number of people affected worldwide totals nearly 44 million [1]. AD results in drastically impaired cognitive function and a reduced capacity to perform even daily routines and activities. Currently, AD diagnosis relies heavily on symptomology with symptom-dependent tools including guidance from the following: (a) National Institute of Neurological and Communicative Disorders and Stroke AD and Related Disorders Association (NINCDS-ADRDA, UK) and (b) Diagnostic and Statistical Manual of the American Psychiatric Association (DSM-IV/DSM-5) [2]. As a result, AD diagnosis is frequently missed or delayed in clinical practice [3]. More recent criteria such as those provided by the National Institute on Aging and the Alzheimer’s Association (NIA-AA) include the use of biomarkers (e.g., β-amyloid and tau) for diagnostic support [4]. As a result, focus has rightly begun to shift toward developing early-stage methods for the detection of possibly potent AD biomarkers. 

Most existing diagnostic methods, e.g., neuroimaging, enzyme-linked immunosorbent assay (ELISA), and polymerase chain reaction (PCR), are not suitable for point-of-care (POC) testing in their current state because they rely on highly sophisticated machinery and equipment, complicated operating procedures, and invasive or destructive sampling methods. Several newer studies have demonstrated greater creativity and have overcome problems by developing new POC testing devices to detect AD-related biomarkers. For example, an electrochemical immuno-sensing approach has been demonstrated for the detection of β-amyloid peptide 1–42 (Aβ42) at pM levels in a relatively shorter period of time than can be accomplished with an ELISA [5]. Stravalaci et al. described a novel immunoassay based on surface plasmon resonance (SPR) that specifically recognizes biologically active oligomers of the β-amyloid peptide (Aβ) [6]. Despite these advances, there is still an urgent need for rapid, effective, and easily used POC devices for early AD screening. The above-mentioned biosensors are not currently practical enough for clinical validation because they may be costly, involve a relatively time-consuming processes (e.g., immunoassay based on SPR requires a 5 h incubation period to produce a maximal signal), or they may require sophisticated signal readers. On the other hand, our paper-based POC device for the detection of Aβ42 is rapid, effective, inexpensive, and requires no sophisticated laboratory equipment. This process relies on an easily accessible body fluid, plasma, that facilitates minimally invasive first-step screening within one and a half hours. 

A paper-based ELISA (P-ELISA) has previously been used to successfully detect proteins such as vascular endothelial growth factor (VEGF), as well as noncollagenous 16A (NC16A) autoimmune antibody toward diagnosis of various diseases such as age-related macular degeneration, bullous pemphigoid and *Escherichia coli* O157:H7 infection [7,8,9,10]. We have now demonstrated a P-ELISA system to detect Aβ42 in plasma. The aim of our study was twofold: (1) to expand the field of biomarker-dependent AD screening, as the use of biomarkers to support diagnosis has gained value and momentum, and, (2) to develop a specific POC tool using a P-ELISA to detect Aβ42 in both buffer and plasma systems. Based on its appropriate limit of detection (LOD), shorter operation duration, and lower cost, this method might set an example for the development of other approaches employing AD-related biomarkers for early stage screening, pre-treatment monitoring, in-treatment monitoring, and post-treatment follow-up. To our knowledge, our study is the first to apply a P-ELISA to detect plasma Aβ42.

Several studies have supported the important role of Aβ42 in the development of AD and have indicated that Aβ42 level dysregulation is responsible for the abnormal accumulation of Aβ42 plaques in the hippocampus and cortex [11,12]. For this reason, Aβ42 has been identified as a diagnostic biomarker, and anti-Aβ-directed therapies have been developed to combat AD [13]. With reliable detection at the core of any diagnostic approach, we first developed a buffer system-based P-ELISA tool to detect Aβ42 in 10-fold dilutions from 1 ng/mL to 1 pg/mL. An outline of our process is provided in Scheme 1 (below). After completing our P-ELISA process (as shown in the supporting movie), we visually interpreted the colorimetric output signal and used a smartphone camera (Apple, 1 Infinite Loop Cupertino, CA 95014, USA) to record the results. This process eliminates the need for any other specialized detector device. Colorimetric assays are particularly well-suited for use in resource-poor settings where plate readers and fluorescence scanners are rare but smartphones are relatively common. We converted our P-ELISA colorimetric results to eight-bit grayscale with ImageJ software using the formula: gray = (red + green + blue)/3. The color intensity was measured from min to max and defined as [experiment zone intensity] − [blank zone intensity]. The Mann–Whitney U test was used to compare the median mean intensity of different Aβ42 concentrations. The LOD was calculated as 63.04 pg/mL, as determined by nonlinear regression fits. Figure 1 displays the significant difference (*p* < 0.001) found between the group with concentrations at 1 ng/mL and our negative control group. The grayscale color intensity values at Aβ42 concentrations of 100, 10, and 1 pg/mL were significantly different (*p* < 0.01) compared to the grayscale color intensity value of the control group. 

Clinically, biomarkers have been used to screen for AD, but these approaches have required semi-invasive cerebrospinal fluid (CSF) sampling via lumbar puncture and/or the use of costly neuroimaging techniques [14]. Transitioning the use of these biomarkers to portable and reliable POC diagnostic devices has been challenging. Cerebrospinal fluid Aβ42 assays may be a more accurate reflection of the central amyloid pathology associated with AD, but there has been some reluctance to employ this approach for routine analysis because of the risk associated with external drains and severe disturbances in CSF [15]. For this reason and others, there have been increased interest and research into the use of more easily accessible sample sources, such as plasma, that contain measurable quantities of Aβ42 suitable for clinical assessment [16]. Previous studies have reported that intra-cerebroventricular injection of Aβ42 is correlated with plasma Aβ42 levels in a mouse model, thus confirming the in vivo mixing of CSF and plasma Aβ42 pools [17]. In humans, a weak positive correlation was also observed between plasma and CSF Aβ42 levels [18]. Moreover, increasing evidence had indicated that plasma Aβ42 concentration may be a risk predictor for AD [19], though some studies have produced controversial results [20]. Kim et al. outlined a filtration-based approach for distinguishing between normal plasma Aβ42 levels and those of patients with AD [21]. Mayeux et al. found mean plasma Aβ42 levels of 82.4 6 ± 8.6 pg/mL among patients with AD and subsequently found baseline mean plasma Aβ42 levels of 68.7 pg/mL and follow-up levels of 76.5 pg/mL in individuals with AD in a later study [22,23]. Using variable capture antibodies and analytical platforms, a wide range of mean plasma Aβ42 levels, from 36 to 140 pg/mL, have been reported in patients suffering from AD [24]. We elected to examine plasma Aβ42 concentration using our own unique P-ELISA approach, employing the same process and equipment employed in our buffer system analysis. We used four sets of plasma samples containing four different concentrations of Aβ42; 0 (control), 10 pg/mL, 100 pg/mL, and 1 ng/mL. For our secondary antibody, we used horseradish peroxidase (HRP) conjugated anti-rabbit antibody (Cat. No.: 7074, Cell Signaling Technology, 3 Trask Lane, Danvers, MA01923, USA) on plasma samples 1 and 2, and we used HRP-conjugated anti-rabbit antibody (Cat. No.: Ab6702, Abcam, Discovery Drive Cambridge Biomedical Campus, Cambridge CB2 0AX, UK) on plasma samples 3 and 4. A comparison between the two secondary antibodies is shown in Table 1. In Figure 2, plasma samples 1 and 2 displayed significant differences (*p* < 0.05) compared to the control for spiked Aβ42 concentrations of 100 pg/mL and 1 ng/mL, respectively. Plasma samples 3 and 4, however, displayed significant differences (*p* < 0.05) compared to the control for spiked Aβ42 concentrations of 10 and 100 pg/mL. From these results, we gathered that secondary antibody selection does appear to affect the performance of our P-ELISA platform. Our plasma system results were also approximately 10 times less sensitive than those from our buffer system. This may be explained by the fact that Aβ42 has to be measured in the matrix as a derivative of blood, which contains very high levels of plasma proteins such as albumin, clotting factor, and immunoglobulin G (IgG), all of which interfere with the application and interpretation of biochemical marker assay results [25,26]. There is room for improvement in the sensitivity and reliability for a plasma-based P-ELISA. Despite these difficulties, a plasma-based P-ELISA system may be used for early AD screening, as suggested by Blennow et al. [27]. Furthermore, repeated longitudinal measurements of plasma Aβ42 level may be useful for routine follow-up to determine disease progression and monitor therapy. 

Clinical AD is thought to be preceded by a long asymptomatic or mildly symptomatic period that may be initiated 15–20 years Fprior to the onset of clinical signs [28]. This pre-dementia period is primarily composed of two parts: (1) preclinical AD and (2) amnestic mild cognitive impairment (MCI) due to AD development (Figure 3). Preclinical AD consists of the following three stages: (1) stage 1, which is manifested by the evidence of amyloidosis; (2) stage 2, which is characterized by not only amyloidosis but also evidence of neurodegeneration; an, (3) stage 3, a combination of amyloidosis, neurodegeneration, and subtle cognitive decline not meeting the criteria for MCI [29]. Compared to preclinical AD, amnestic MCI due to AD is defined as noticeable cognitive impairment resulting from underlying AD pathology. Because the development of AD is irreversible and progressive, there is an increasing need for biomarker-based screening tools to identify patients in preclinical or early clinical stages of AD. These patients would be greatly benefited by early intervention before more severe and irreversible damage occurs to the brain. In the past decade, a number of studies have made great efforts to develop biomarker-based screening tools and POC testing platforms to diagnose AD. Nakamura et al. validated the clinical utility of a blood-based Aβ assay using immunoprecipitation and mass spectrometry to predict brain Aβ burden [30]. Garyfallou et al. demonstrated an electrochemical immunosensor that can be easily integrated into portable devices to diagnose AD using plasma immunoglobulins [31]. Tonello et al. developed a POC testing system based on screen-printed electrochemical sensors (SPES) [32]. This study, however, is the first to demonstrate a P-ELISA system for Aβ42 detection in human plasma. It is challenging to measure Aβ42 due to antibody masking, Ab oligomerization, and Ab complex formation [33]. Plasma Aβ42 is also hard to use for diagnosing late-onset AD as a single time-point measure due to the considerable overlap with changes in the normal, aging population and the onset of vascular diseases [18,34]. We hope to promote the use of a P-ELISA for early screening, routine follow-up analyses, as well as AD monitoring in living patients as an adjunct to care. If detected at concentrations associated with risk, Aβ42 levels can be modified, as demonstrated by Boada et al., who describe a process for modifying Aβ42 concentration in plasma using plasma exchange (PE) and albumin replacement that improved cognition in patients with mild-to-moderate AD [35]. Our P-ELISA platform can help optimize therapeutics and improve disease progression prediction [36]. P-ELISA methods provide several advantages compared to conventional ELISA methods (Table 2). First, the entire P-ELISA process, from antigen immobilization to colorimetric reaction, can be completed within one and half hours; by contrast, a conventional ELISA requires at least six-to-eight hours to complete. Second, a P-ELISA requires only 3 μL of sample per test zone, while conventional ELISA requires more than twenty-five times that. Finally, P-ELISA results can be quantified with simple devices, such as smartphone cameras, which increases their usability and broadens their impact. Further research could result in the production of a paper-based multiplexed assay incorporating peptide-detecting ELISA to create a multi-step, all-in-one diagnostic device [37,38]. We have accomplished the first step toward this goal, creating a paper-based device for peptide detection, with this study.

This study outlines our development of the first P-ELISA tool for Aβ42 detection with demonstrated potential for testing human plasma. Our findings underscore the potential for employing a P-ELISA for both pre-clinical AD screening and post-diagnosis treatment monitoring. Compared to commonly-used Aβ42 detection methods, the P-ELISA offers five principal advantages: 1) rapidity, 2) small sample and reagent volume requirements, 3) cost-effectiveness, 4) readily available equipment and materials, and 5) improved clinical safety due to the fact that required samples involve the appropriation of plasma as opposed to CSF via lumbar puncture. P-ELISA techniques require some improvement in accuracy, precision, and long-term stability to render them more commercially viable. However, we found our approach to be highly sensitive, as evidenced by the 63.04 pg/mL LOD value attained in our buffer system experiments. In conclusion, our P-ELISA system is a promising candidate for the early screening of AD pre-dementia period and the post-diagnostic monitoring of AD, especially in small laboratories and in developing countries where cost and convenience are more critical.

## Figures and Tables

**Figure 1:**
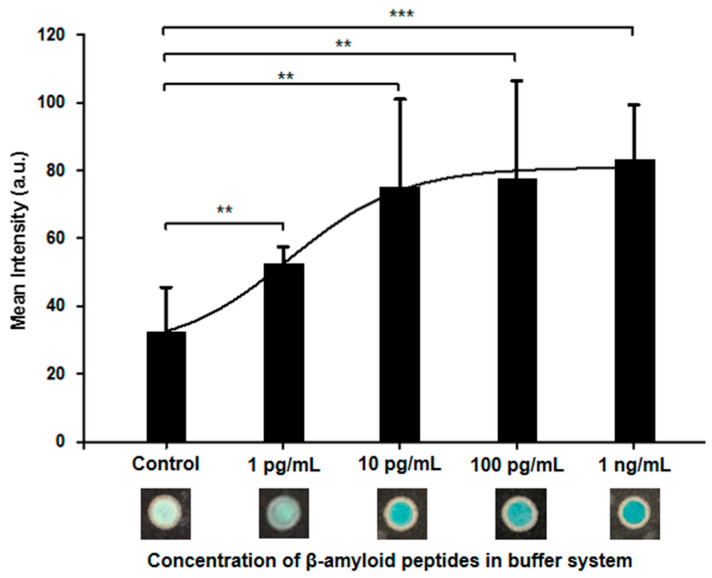
Colorimetric results (intensity) from our paper-based ELISA (P-ELISA) test for β-amyloid peptide 1-42 (Aβ42) concentrations in a buffer system. The color intensity difference between our 1 pg/mL Aβ42 concentration and our control was very significant. (** *p* < 0.01; *** *p* < 0.001).

**Scheme 1:**
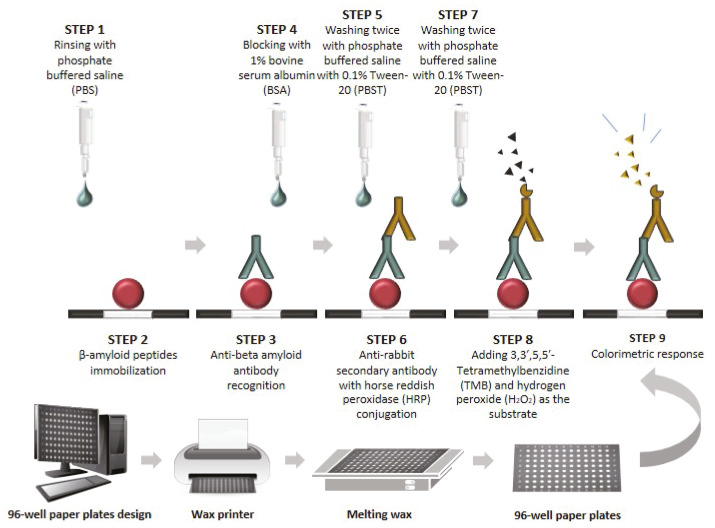
Schematic of our paper-based ELISA (P-ELISA) device development and test procedure for the detection of β-amyloid peptide 1-42 (Aβ42) concentrations in both buffer and plasma systems.

**Figure 2:**
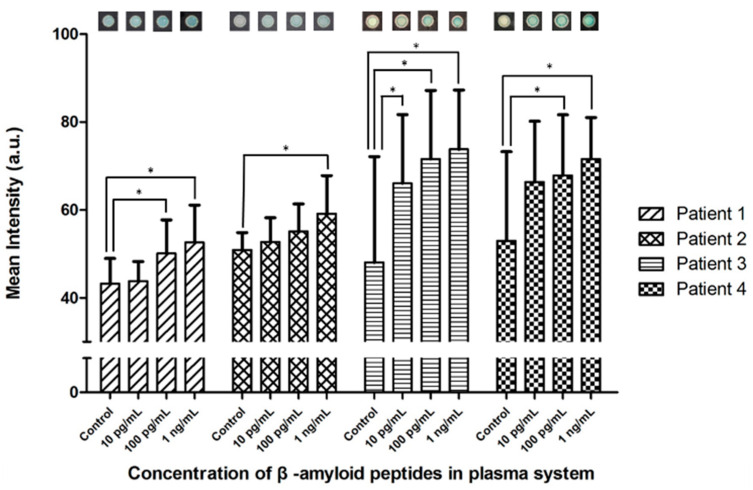
Colorimetric results (intensity) from a paper-based ELISA (P-ELISA) test for β-amyloid peptide 1–42 (Aβ42) concentration in a plasma system. The secondary antibody used in plasma 1 and 2 was from Cell Signaling Technology, while that used in plasma 3 and 4 was from Abcam. The limit of detection (LOD) for tests using plasma 1 and 2 was approximately 100 pg/mL, while the LOD for tests using plasma 3 and 4 was about 10 pg/mL. (* *p* < 0.05).

**Figure 3:**
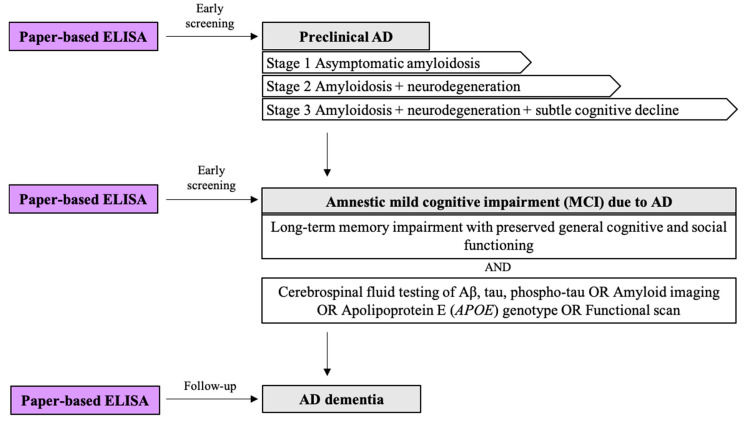
The role of point-of-care (POC) β-amyloid peptide 1–42 (Aβ42) testing for patients with preclinical Alzheimer’s disease (AD), amnestic mild cognitive impairment (MCI) due to AD, and AD dementia.

**Table 1:** Comparison between the two secondary antibodies used in our paper-based ELISA (P-ELISA) system for the detection of β-amyloid peptide 1–42 (Aβ42).

	Goat Anti-Rabbit IgG H and L (Cat. No.: Ab6702)	Anti-Rabbit IgG, HRP-Linked Antibody (Cat. No.: 7074)
Host Species	Goat	Goat
Target Species	Rabbit	Rabbit
Clonality	Polyclonal	Polyclonal
Isotype	IgG	IgG
Performance	10 pg/mL	100 pg/mL
Brand	Abcam	Cell Signaling Technology

**Table 2:** Comparison between the paper-based ELISA (P-ELISA) and conventional enzyme-linked immunosorbent assay (ELISA) systems for the detection of β-amyloid peptide 1–42 (Aβ42) using plasma and cerebrospinal fluid (CSF) samples.

	Paper-based ELISA (P-ELISA)	Enzyme-Linked Immunosorbent Assay (ELISA) [25,39]	
**Time**	1.5 h	6–8 h (at least)	
Sample Volume (per Test Zone)	3 μL	75 μL	100−370 μL
Sample Source	Buffer	Plasma	CSF
Limit of Detection	63.04 pg/mL	5.71 pg/mL	312 pg/mL

## Supplementary Materials

Supplementary File 1

## Abbreviations

**AD**	Alzheimer’s disease
**P-ELISA**	Paper-based ELISA
**Aβ42**	β-amyloid peptide 1–42
**MCI**	Mild cognitive impairment
**NINCDS-ADRDA**	National Institute of Neurological and Communicative Disorders and Stroke AD and Related Disorders Association
**DSM**	Diagnostic and Statistical Manual of the American Psychiatric Association
**NIA-AA**	National Institute on Aging and the Alzheimer’s Association
**ELISA**	Enzyme-linked immunosorbent assay
**PCR**	Polymerase chain reaction
**POC**	Point-of-care
**SPR**	Surface plasmon resonance
**Aβ**	ββrface plpeptide
**VEGF**	Vascular endothelial growth factor
**NC16A**	Noncollagenous 16A
**LOD**	Limit of detection
**CSF**	Cerebrospinal fluid
**HRP**	Horseradish peroxidase
**IgG**	Immunoglobulin G
**SPES**	Screen-printed electrochemical sensors
**PE**	Plasma exchange
***APOE***	Apolipoprotein E
